# Presepsin as a Diagnostic and Prognostic Biomarker in Sepsis

**DOI:** 10.7759/cureus.15019

**Published:** 2021-05-13

**Authors:** Dimitrios Velissaris, Nicholas Zareifopoulos, Vasileios Karamouzos, Evangelos Karanikolas, Charalampos Pierrakos, Ioanna Koniari, Menelaos Karanikolas

**Affiliations:** 1 Department of Internal Medicine, General University Hospital of Patras, Patras, GRC; 2 Department of Psychiatry, General Hospital of Nikaia, Piraeus "Agios Panteleimon", Athens, GRC; 3 Department of Internal Medicine, University of Patras School of Health Sciences, Patras, GRC; 4 Intensive Care Unit, General University Hospital of Patras, Patras, GRC; 5 Department of Internal Medicine, University of Patras, Patras, GRC; 6 Intensive Care Unit, Brugmann University Hospital, Université Libre de Bruxelles, Brussels, BEL; 7 Department of Electrophysiology and Device, University Hospital of South Manchester NHS Foundation Trust, Manchester, GBR; 8 Department of Anesthesiology, Washington University School of Medicine, St. Louis, USA

**Keywords:** cd14, sepsis, lps, systemic inflammatory response syndrome, gram-negative bacteremia, chronic renal diseases, mortality rate in icu, shock, procalcitonin, crp levels

## Abstract

Sepsis is a condition characterized by high morbidity and mortality which is commonly encountered in an emergency and critical care setting. Despite a substantial body of research, the ideal biomarker for the diagnosis and prognostic stratification of septic patients remains unknown. This review aimed to summarize the publications referring to the validity of the biomarker presepsin when used for the detection, monitoring and prognosis in patients suffering with sepsis.

This work is a narrative review based on a PubMed/Medline search conducted in order to identify all relevant publications referring to the use of presepsin in sepsis. Search was not limited by year of publication so all articles archived in the database would be retrieved. No article from before 2010 was identified.

A total of 57 publications of the last decade were included, all of which support the use of presepsin as a biomarker for the assessment of septic patients. It has been used alone or in combination with commonly used biomarkers in the evaluation of patients with sepsis in settings such as the emergency department and the intensive care unit. It is useful in the initial workup of patients with suspected sepsis in the emergency setting and may be a predictive factor of mortality and the most severe complication of sepsis.

Presepsin seems to be a valuable tool for the laboratory workup of sepsis, especially when used in conjunction with other biomarkers and clinical rating scores with an established role in this population. Further research is needed to evaluate the clinical implications of utilizing presepsin measurements in the workup of sepsis.

## Introduction and background

Sepsis is among the most common causes of death for hospitalized patients and despite recent progress mortality rates remain unacceptably high, especially if septic shock is present. The financial burden of the syndrome is significant as it commonly requires prolonged treatment in the intensive care unit [1]. The pathogenesis of the syndrome involves a complex interplay between the pathogen (typically gram-negative bacteria) and the host immune response. Several biomarkers have been used in clinical practice for better monitoring, management and risk stratification of the sepsis syndrome. Presepsin is an immunologic biomarker which has been identified during the past decade as a new, emerging, early indicator for the detection of infections [2].

Presepsin (sCD14-ST), which is ∼13 kDa in size, is a soluble N-terminal fragment of the cluster of differentiation marker protein CD14, which is a soluble form of the lipopolysaccharide (LPS) receptor, a member of the family of toll-like receptors which recognize pathogen-associated molecular patterns (PAMPs) and initiate the innate immune response [3]. LPS is a component of the gram-negative bacterial cell wall which strongly stimulates innate immunity, thus contributing to the pathogenesis of sepsis. CD14 is a transmembrane protein present in many cells implicated in the sepsis cascades, including macrophages, monocytes, and granulocyte cells which is responsible for the intracellular transduction of endotoxin signals [3]. During the process of the inflammation, soluble CD14 fragments (presepsin) are cleaved which can be readily measured using a chemiluminescent enzyme immunoassay [2]. Because presepsin is elevated early during sepsis and is relatively specific for bacterial infection (due to its role in the pathogenesis of sepsis as a receptor for LPS), it may be useful in a clinical setting as a biomarker for the diagnosis and risk stratification of patients who are suspected to be septic. Compared to other biomarkers used for this purpose (procalcitonin, C-reactive protein) it is hypothesized to be more specific for sepsis, as it is directly implicated in the pathogenesis of the syndrome. Several studies have investigated the validity of presepsin when used in clinical practice [4]. It should be noted that presepsin measurement is not ubiquitously available yet unlike other biomarkers of sepsis. Its role as a diagnostic and prognostic biomarker for the risk stratification of sepsis is further evaluated in clinical settings including the Emergency Department and the Intensive Care Unit. The promising role of presepsin was reported in the review by Pizzolato in 2014, as it was noted that presepsin had a high sensitivity and good specificity for sepsis, is readily available in the emergency department and correlated to in-hospital mortality in patients with sepsis and septic shock. It was noted that presepsin levels may have similar specificity and sensitivity to clinical rating systems such as the MEDS and SOFA scores, the diagnostic and accuracy of which may be substantially improved if they are combined with presepsin and other biomarkers [4]. The promising role of presepsin as a new biomarker for early diagnosis of sepsis was identified in another review published the same year [5]. The authors noted that presepsin may have better prognostic validity than procalcitonin, C-reactive protein (CRP) and the erythrocyte sedimentation rate (ESR), while being more specific for infection compared to both lactate and the ESR [4]. Herein we present a review of all identified bibliography which is related to the use of presepsin in sepsis.

## Review

Methods

**Figure 1:**
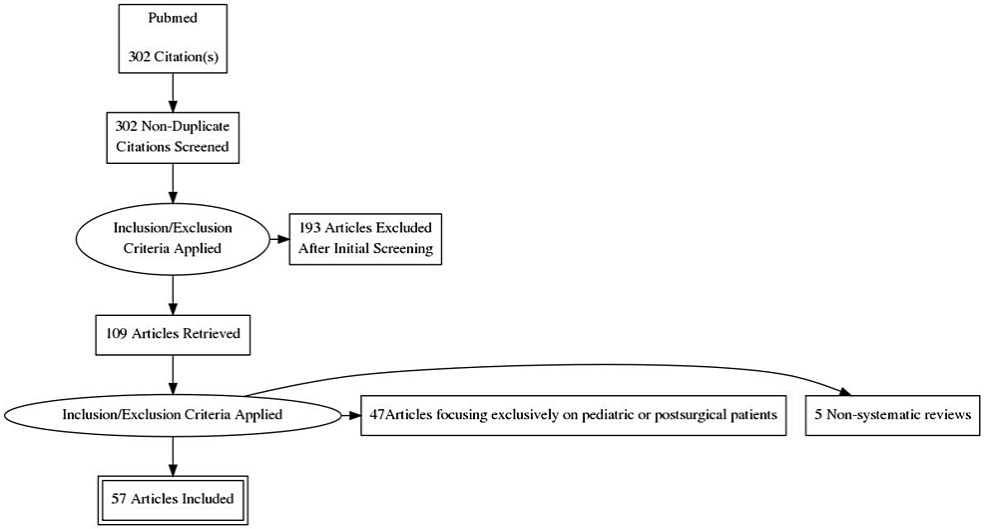
Review flow chart

A PubMed search was conducted on December 2020 using the terms ‘presepsin in sepsis’ as “Title/Abstract” or as “MeSH Terms”. The structure of the search in the “Search details” window of the PubMed website was presepsin [All Fields] AND ("sepsis"[MeSH Terms] OR "sepsis"[All Fields]). The literature search was limited to articles referring only to adult patients and the extracted bibliography was further searched for more related publications, by hand search of the references of retrieved articles. Studies focusing exclusively on postoperative patients were excluded as the value of presepsin measurement in this population would best be evaluated separately, with studies grouped based on the type of surgical procedure. Only manuscripts written in the English language were included in this review. Though this work is a narrative review, a PRISMA flowchart is provided (Figure 1).

Results

Presepsin as a Diagnostic Marker of Infection

Several studies have evaluated the use of presepsin measurements to diagnose infection during the initial presentation of the patient. It is hypothesized that it may be the ideal biomarker for diagnosing bacterial infection as it is directly implicated in the innate immune response and its concentration rises within hours of infection. This has been evaluated in a few systematic reviews and meta-analyses apart from original studies. A meta-analysis published in 2015 evaluated presepsin for the diagnosis of sepsis based on publications retrieved from medical databases until November 7, 2014. Eleven studies were finally included in this meta-analysis. This systematic review showed that presepsin was an effective adjunct biomarker for sepsis diagnosis, but insufficient to detect or rule out sepsis when used alone [6]. Another meta-analysis published also in 2015, based on PubMed, Embase, Web of Science and Cochrane databases included eight studies with a total of 1,815 patients. It was reported that presepsin exhibits a very good diagnostic accuracy (Area under the curve, AUC=0.89) for the diagnosis of sepsis [7]. The same year another meta-analysis by Wu et al. was published based on database search up to 15 December 2014. Nine studies with 10 trials and 2159 cases were included. It was demonstrated that presepsin had some superiority in the management of patients, and could be a helpful biomarker in sepsis early diagnosis. However, presepsin showed a moderate diagnostic accuracy in differentiating sepsis from non-sepsis cases [8]. In 2015 another systematic review was published, a bivariate meta-analysis based on data from PubMed and EMBASE which included 11 publications with 3,106 subjects. Presepsin found to have a valuable role in the diagnosis of sepsis, but the results should be interpreted carefully in clinical practice and in comparison to the traditional markers [9].

One of the first clinical applications of presepsin measurement that was investigated was its use for the initial diagnosis of infection upon patient presentation to the emergency department. For this purpose presepsin could be used alone or in conjunction with other biomarkers commonly employed for this purpose, such as procalcitonin (PCT) and C-reactive protein (CRP). Based upon the available evidence presepsin is comparable in sensitivity to the aforementioned biomarkers and more specific compared to complete blood count abnormalities such as increased white blood cell count [10], though it may not offer any significant advantages over PCT, which is more widely available [11]. The sensitivity of presepsin (approximately 95% with a cutoff value of 729 pg/mL for a positive test [12]) may be sufficient to rule out infection, but it must be used in conjunction with other biomarkers and clinical scoring systems to definitively rule in infection. Presepsin values have a strong positive correlation with the CRP, PCT and the SOFA and MEDS scores [13]. Higher values of presepsin are associated with septic shock compared to severe sepsis without shock, a trend similar to that observed with PCT [1]. In probable infections with an identifiable focus, presepsin may be of value in differentiating whether an exacerbation of chronic obstructive pulmonary disease is due to pneumonia or non-infectious in etiology [14], and may also be useful in patients presenting with nephrolithiasis and systemic inflammatory response syndrome (SIRS) symptoms to rule out pyelonephritis as a cause [15], and may aid in differentiating between the causes of acute abdomen [16]. In patients with definite pyelonephritis, a higher presepsin cutoff may be used to test for the presence of bacteremia [17]. A similar effect was found in a study of burn patients, who are at increased risk for severe disseminated infection [18].

In hospitalized patients or individuals already being treated in an ICU setting presepsin may retain its utility for the diagnosis of emerging infections. In an ICU setting it may be comparable to other emerging biomarkers, including pro-adrenomedullin [19] and soluble triggering receptor expressed on myeloid cells-1 (sTREM-1) [20], and a combination of these biomarkers may be superior for the diagnosis of sepsis compared to either used alone. In this it could also be useful for the differential diagnosis of acute respiratory distress syndrome (ARDS), as it appears to be elevated only in ARDS due to sepsis and not in non-infectious causes of the syndrome [21]. Outside of the ICU, presepsin may be useful to differentiate sepsis from non-infectious SIRS with accuracy comparable to procalcitonin [22-24]. It should be noted that although its biological function is mostly associated with the response to gram negative bacteremia, an early study found presepsin measurements did not differentiate gram negative and gram positive bloodstream infection [25]. Presepsin was found to be an accurate biomarker for sepsis in geriatric individuals over 75 years of age in combination with PCT and the early warning score [26]. It was also useful for the diagnosis of bacteremia in hematological patients undergoing stem cell transplant with a cutoff value of 218 pg/ml [27] and in another study of hematological patients undergoing chemotherapy was found useful for the differentiation between bacterial and fungal infection [28]. This study confirmed the relative specificity of presepsin for bacterial infection and the authors that elevated CRP in conjunction with presepsin within the normal reference range was in favor of fungal infection in this group of immunocompromized individuals. A summary of the studies on the diagnostic role of presepsin can be found in Table 1.

**Table 1:** Presepsin as a diagnostic marker of infection APACHE II: Acute physiology and chronic health evaluation II, ARDS: Acute respiratory distress syndrome, CAP: Community acquired pneumonia, CD64: Cluster of differentiation 64, COPD: Chronic obstructive pulmonary disease, CR: Clearance ratio, CRP: C-reactive protein, DIC: Disseminated intravascular coagulation, DNA: Deoxyribonucleic acid, ED: Emergency department, EMBASE: Excerpta medica database, hsCRP: high sensitivity C-reactive protein, HSCT: Hematopoietic stem cell transplantation, ICU: Intensive care unit, IL: Interleukin, MEDLINE: Medical Literature Analysis and Retrieval System Online, MEDS: Mortality in emergency department sepsis, MR-proADM: mid-regional pro-adrenomedullin, PCT: Procalcitonin, pro-ADM: Proadrenomedullin, ROC: Receiver operating characteristic, sTREM-1: soluble triggering receptor expressed on myeloid cells-1, SCD14-ST: soluble Cluster of Differentiation 14 subtype, SIRS: Systemic inflammatory response syndrome, SOFA: Sequential Organ Failure Assessment, WBC: White blood cell.

1^st^ Author and year of publication	Study design	Type of patients/database	Major findings
Halıcı [14], 2020	Prospective cohort study	126 patients admitted for COPD exacerbation in a single tertiary center	Presepsin at admission was accurate for diagnosing pneumonia as a cause of acute COPD exacerbation, but a positive associated between elevated presepsin and mortality did not reach statistical significance.
Chen [20], 2020	Prospective case-control	60 patients admitted to a single ICU with sepsis and 60 matched controls	Presepsin and the biomarker sTREM-1 were more sensitive for the diagnosis of sepsis in the ICU compared to procalcitonin and CRP, with the most accurate indicator being a composite biomarker of presepsin and sTREM-1. In patients diagnosed with sepsis, higher values of both biomarkers were associated with mortality. A cutoff value of 1025 pg/ml presepsin had 83% specificity and 85% sensitivity for the diagnosis of sepsis.
Ruangsomboon [26], 2020	Prospective cohort	Single center study of 250 patients > 75 years of age admitted on suspicion of sepsis	Presepsin had a similar diagnostic and prognostic accuracy as procalcitonin and the early warning score. The combination of the three biomarkers was superior to the use of either alone, and may be useful for the timely diagnosis of sepsis in the geriatric population.
Stoma [28], 2019	Prospective	Hematological patients with sepsis	This prospective study enrolled 64 hematological hospitalized patients to receive chemotherapy. These had proven or probable invasive fungal infection or microbiologically proven bacterial bloodstream infection. In total, 53 patients with bacterial bloodstream infections and 11 with invasive fungal infections participated in the study. Results showed that a combination of CRP >120 with PCT <1.25 or presepsin <170 could be a possible combined biomarker for invasive fungal infections in immunocompromised patients.
Lu [10], 2018	Prospective cohort study	Patients with sepsis and SIRS in the ED	The values of presepsin, PCT, CRP and WBC were evaluated in patients with sepsis and SIRS when assessed in the Emergency Department in the study by Lu. 72 patients with sepsis and 23 patients with non-infectious SIRS were enrolled. The levels of the above biomarkers were apparently higher in sepsis patients than in the non-bacterial SIRS group (P < 0.05). Also, the levels of presepsin and the APACHE II score demonstrated a significant difference among sepsis, severe sepsis and septic shock group of patients (P < 0.05). The authors concluded that presepsin was a promising biomarker for initially diagnosis and risk stratification of sepsis, also a potential marker to distinguish gram-positive and gram-negative bacterial infection.
Sargentini [19], 2017	Prospective case-control study	64 ICU patients	64 ICU patients were enrolled in this case-control study, where values of inflammatory biomarkers PCT, presepsin and pro-ADM were evaluated. These biomarkers were significantly lower in controls than in sepsis or septic shock groups. Preliminary data showed that, despite presepsin and pro-ADM being able to differentiate between septic and non-septic patients with accuracy, PCT remains the most reliable marker available.
Claessens [17], 2017	Prospective cohort study	312 patients with acute pyelonephritis and bacteremia	Values of presepsin were assessed in patients with acute pyelonephritis and controls, and their capacity to predict bacteraemia on admission was evaluated. In 312 patients with acute pyelonephritis, presepsin concentrations were higher than in controls, and increased in patients with bacteraemia and in those requiring admission. Performance of presepsin to predict bacteraemia was similar to CRP and less accurate than PCT. Although presepsin seems to be a valuable biomarker to detect patients with acute pyelonephritis did not offer advantage when comparing to CRP and PCT due to mild prediction of bacteraemia and need for admission.
de Guadiana Romualdo [11], 2017	Prospective cohort study	223 patients with suspected sepsis in the ED	The diagnostic accuracy of presepsin for infection and sepsis, compared with PCT and CRP was evaluated in the emergency department (ED) setting. 223 patients with suspected infection were enrolled in the study. Results showed that median CRP, PCT and presepsin levels were significantly higher in patients with infection and sepsis. PCT had the highest performance for infection and for sepsis, PCT and presepsin performed a similar one. The diagnostic accuracy of presepsin in this study does not improve that of PCT.
Stoma [27], 2017	Observational, prospective study	52 neutropenic patients after hematopoietic stem cell transplantation (HSCT)	A prospective observational study based on data from 52 neutropenic patients after hematopoietic stem cell transplantation (HSCT) was conducted in order to assess the diagnostic values of presepsin, PCT, and CRP in these patients in a condition of high prevalence of gram-negative pathogens. Results showed the best diagnostic value for presepsin and this biomarker may be recommended in adult patients with suspected gram-negative blood stream infection after HSCT as a possible additional supplementary test with a cut-off value of 218 pg/mL
Ali [22], 2016	Prospective cohort study	51 patients with SIRS and suspected sepsis, 25 healthy controls	Plasma presepsin, PCT and CRP levels in patients with SIRS and suspected sepsis were serially measured in order to evaluate the diagnostic and prognostic performance of presepsin in comparison to PCT and CRP. Results of the study showed that presepsin and PCT yielded similar diagnostic accuracy, whereas presepsin performed significantly better than CRP. Presepsin and PCT had comparable accuracy in differentiating between septic and non-septic patients. Finally, early changes in presepsin concentrations might reflect the appropriateness of the therapeutic modality.
Leli [23], 2016	Prospective cohort study	92 patients with suspected sepsis	The diagnostic accuracy of presepsin in predicting bacteraemia and bacterial DNAaemia in patients with suspected sepsis, and its comparison with that of PCT and CRP was assessed in this study. The presepsin median values were significantly higher in bacteraemic vs non-bacteraemic patients and in patients with bacterial DNAaemia vs patients without. This study concluded that when sepsis is suspected, presepsin and PCT had a good diagnostic accuracy in predicting both bacteraemia and bacterial DNAaemia, superior to CRP.
Zhang [6], 2015	Meta-analysis	11 studies	Presepsin is an effective adjunct biomarker for sepsis diagnosis, but insufficient to detect or rule out sepsis when used alone.
Zhang [7], 2015	Meta-analysis	8 studies with a total of 1,815 patients	Presepsin exhibits a very good diagnostic accuracy for diagnosing sepsis.
Wu [8], 2015	Meta-analysis	9 studies with 10 trials and 2159 cases	Presepsin had some superiority and may be a helpful biomarker in sepsis early diagnosis.
Tong [9], 2015	Bivariate meta-analysis	11 publications with 3,106 subjects	Valuable role in the diagnosis of sepsis, but should be interpreted carefully in clinical practice and in comparison to traditional markers.
Hou [15], 2015	Prospective cohort study	39 patients with nephrolithiasis presenting with SIRS	A prospective study aimed to evaluate the diagnostic ability of presepsin in the differential diagnosis between SIRS, infection, or sepsis and to compare its diagnostic value with other used biomarkers (CRP, PCT, and WBC) in patients of nephrolithiasis presenting with SIRS. 39 patients were enrolled in the study and the plasma presepsin was detected by the Pathfast presepsin assay system. ROC analysis showed that presepsin had a high diagnostic value compared with both PCT and CRP. In the early stage of SIRS, presepsin remained a highly sensitive and specific diagnostic marker compared with either PCT, CRP, or WBC.
Kweon [24], 2014	Prospective cohort study	118 patients presenting to the ED with suspected sepsis	Higher diagnostic accuracy of presepsin compared to other conventional biomarkers
De Guardiana-Romualdo [12], 2014	Prospective cohort study	226 patients with SIRS in the ED	226 patients with SIRS who were admitted in the Emergency Department assessed in an attempt to compare the validity of presepsin with the commonly used available sepsis biomarkers. The results showed that presepsin values were significantly higher in the SIRS group with bacteremia compared to the non-bacteremic SIRS group. The best cut-off value was 729 pg/mL and this was associated with a negative predictive value of 94.4%. It was concluded that presepsin may contribute to rule out the diagnosis of bacteremia in SIRS patients in the ED setting.
Cakır Madenci [18], 2014	Prospective cohort study	37 sepsis patients in a burn center	The validity of presepsin was evaluated in the diagnosis and follow up of sepsis in burn patients. A prospective study in a burn center included 37 patients. Presepsin, PCT, CRP and WBC levels were measured. Patients were classified as sepsis or non-sepsis according to the American Burn Association Consensus Criteria (ABA) 2007. The authors concluded that presepsin plasma levels had comparable performance in burn sepsis.
Liu [13], 2013	Prospective cohort study	859 patients with at least 2 diagnostic criteria for SIRS in the ED	A prospective study conducted in the ED enrolled 859 patients with at least 2 diagnostic criteria for SIRS. The aim was to evaluate the early diagnostic and prognostic value of presepsin compared with PCT, mortality in Emergency Department Sepsis (MEDS) score and APACHE II score in septic patients in the ED setting. Plasma presepsin levels in septic patients were significantly higher in non-survivors at 28 days' follow-up. Presepsin and both MEDS and APACHE II scores were independent predictors of severe sepsis, septic shock and 28-day mortality in septic patients. Levels of plasma presepsin were positively correlated with PCT, MEDS score and APACHE II score in every septic group, thus was considered a valuable marker for early diagnosis of sepsis and risk stratification.
Ulla [1], 2013	Case-control study	106 patients in the ED with suspected sepsis/septic shock, and 83 patients with SIRS but without clinical evidence of infection	In 2013 a two-center study was published based on 106 patients who presented in the ED with suspected sepsis or septic shock, and another 83 patients affected by SIRS but without clinical evidence of infection. The blood samples of the patients were analyzed using the PATHFAST presepsin assay for sCD14. In septic patients it was observed elevated presepsin concentrations on admission compared to controls. The same trend was observed for mean values of PCT. Mean presepsin values were significantly higher in non-survivor septic patients than in survivors. On the contrary, no significant correlation was noted between PCT and survival. Presepsin had a significant prognostic value in the population assessed in the ED.
Vodnik [16], 2013	Prospective cohort study	70 patients with bacterial and non-bacterial infection in acute abdominal conditions	The clinical usefulness of presepsin in the differentiation between bacterial and non-bacterial infection in acute abdominal conditions was reported in this study. The presepsin values were significantly higher in patients with sepsis than the SIRS patients. It was concluded that presepsin was a significantly sensitive indicator of sepsis and a useful biomarker for the rapid diagnosis of sepsis.
Endo [25], 2012	Multi-center prospective cohort study	207 patients with bacterial and non-bacterial infections	207 patients were enrolled in a multi-center prospective study which aimed to assess the clinical usefulness of presepsin in bacterial and non-bacterial infections. Levels of presepsin, PCT, and IL-6 were significantly higher in patients with bacterial infectious disease. Presepsin levels did not differ significantly between patients with gram+ and gram- bacterial infections. The usefulness of presepsin for diagnosing sepsis was concluded, also presepsin possible superiority to conventional biomarkers and blood culture.

Presepsin as a Prognostic Indicator in Sepsis

Similarly to other biomarkers of infection, serial presepsin measurements may be useful for the prognostic stratification of septic patients. Higher measurements may be associated with greater mortality and increased risk of the severe complications of sepsis that are typically encountered in an ICU setting, specifically acute renal failure, septic shock, acute respiratory distress syndrome (ARDS) and disseminated intravascular coagulation (DIC). Two meta-analyses which evaluated the prognostic role of presepsin in septic patients have been published, one in a critical care setting [29] and the other upon admission to the hospital [30]. By comparing the population of survivors with non-survivors both found consistently elevated presepsin levels in non-survivors, suggesting that presepsin elevation reflects mortality risk and may be useful as an independent predictor of mortality, thus aiding in the identification of patients most likely to deteriorate.

Serial presepsin measurements may be used to evaluate changes in a patient’s condition in the intensive care setting. The first few days after ICU admission are crucial, as that is when the most severe complications of sepsis typically develop. A recent study indicated that presepsin elevation upon ICU admission and on day 2 was a prognostic factor for acute renal failure, presepsin on days 1-3 as well as a composite factor comprising of presepsin elevation and the Glasgow prognostic score predicted the development of ARDS, whereas presepsin elevation on the first two days of ICU admission predicted the development of DIC [31], findings which may be of great interests to intensivists should presepsin measurements become more widely available. Presepsin elevation also predicts mortality in sepsis-induced ARDS [21]. In the same vein, a marked reduction in presepsin levels may foreshadow clinical improvement, indicating response to treatment [32]. It is clear from these results that if serial presepsin measurements are obtained a downtrend is a good prognostic indicator while an uptrend is indicative of a worse prognosis and a more complicated clinical course [21, 32].

Several studies in an ICU compared the prognostic utility of presepsin to other commonly used biomarkers and clinical rating scales, including PCT, lactate, CRP, proadrenomedullin, gelactin-3 [33], and the APACHE, MELD and SOFA scores. Presepsin consistently outperformed CRP [34,35] but was not characterized by any significant advantage in comparison to PCT or the clinical rating scores that were examined, though its value may lie in the utility of its combination with other biomarkers [33,36,37]. Presepsin levels correlate significantly with both PCT and the SOFA score and are an independent predictor of mortality in patients treated in the ICU for sepsis [38-41], and similar findings were also observed in cohorts of hospitalized patients not in intensive care [42,43]. Only in one cohort were there no promising results [44] at all in the comparison between presepsin, PCT, CRP and lactate. One particularly interesting article reported on the prognostic value of the clearance ratio of presepsin as ascertained by serial measurements which was superior to the clearance ratio of PCT as a predictor of mortality in inpatients with severe sepsis, which warrants further investigation [45]. One study found a sustained elevation of presepsin with simultaneous normalization of PCT predicted relapse of sepsis, though further research is required to evaluate whether these findings are reproducible [37].

Presepsin may also retain a significant predictive value in the emergency department setting, where initial elevation may be a significant predictor of early mortality due to sepsis [46,47]. One study which evaluated it along CRP, interleukin-6 and PCT reported that presepsin was the only biomarker that remained elevated after treatment initiation in the group with the most severe initial presentation and retained an association with an elevated risk of mortality throughout the follow-up period [48]. Another study found presepsin and CRP elevation in the emergency room to be predictive factors of DIC, with the combination of both having a stronger association with an increased risk of DIC than either alone [49]. These findings suggest it may be of superior prognostic value compared to other commonly used biomarkers in the emergency setting, though further research is required to evaluate these associations.

The prognostic utility of presepsin has also been evaluated in hospitalized patients with a known cause of infection. Two studies on patients with confirmed pneumonia noted an association between presepsin elevation and mortality, with presepsin elevation being more pronounced in septic patients compared to those with the relatively milder presentation of severe pneumonia [50,51]. Similar findings were also reported in a cohort of patients with another definite focus of infection, an enterocutaneous fistula [52]. The authors concluded that presepsin seems to have prognostic values for source control of abdominal sepsis⁠. A single prospective study evaluated whether presepsin elevation could be predictive of mortality in disseminated fungal infection with fungemia as it is in bacterial sepsis. The authors noted pronounced presepsin elevation with normal procalcitonin, challenging the notion that presepsin is in fact specific for bacterial infection [53]. The degree of presepsin elevation appeared to correlate with clinical severity and mortality in patients with disseminated fungemia. A summary of studies on presepsin as a prognostic indicator in sepsis can be found in Table 2.

**Table 2:** Presepsin as a prognostic marker in sepsis APACHE II: Acute physiology and chronic health evaluation II, ARDS: Acute respiratory distress syndrome, CAP: Community acquired pneumonia, CR: Clearance ratio, CRP: C-reactive protein, DIC: Disseminated intravascular coagulation, ED: Emergency department, EMBASE: Excerpta medica database, hsCRP: high sensitivity C-reactive protein, ICU: Intensive care unit, IL: Interleukin, MEDLINE: Medical Literature Analysis and Retrieval System Online, MEDS: Mortality in emergency department sepsis, MR-proADM: mid-regional pro-adrenomedullin, PCT: Procalcitonin, SCD14-ST: soluble Cluster of Differentiation 14 subtype, SIRS: Systemic inflammatory response syndrome, SOFA: Sequential Organ Failure Assessment, WBC: White blood cell.

1^st^ Author and year of publication	Study design	Type of patients/database	Major findings
Shimoyama [31], 2021	Prospective cohort study	Single center ICU, 83 patients	Presepsin elevation upon ICU admission and on day 2 was a prognostic factor for acute renal failure, presepsin on days 1-3 as well as a composite factor comprising of presepsin elevation and the Glasgow prognostic score predicted the development of ARDS, whereas presepsin elevation on the first 2 days of ICU admission predicted the development of DIC.
Aliu-Bejta [42], 2020	Prospective cohort study	100 patients admitted with sepsis in 2 tertiary care centers	Presepsin levels at baseline were significantly elevated in patients with septic shock compared to patients with simple sepsis according to the sepsis-3 definition. Presepsin correlated strongly with the SOFA score. CRP and procalcitonin elevation did not differentiate between sepsis and septic shock.
Zhao [21], 2020	Prospective cohort study	Multicenter study of 225 patients with ARDS	Presepsin elevation enabled the discrimination of ARDS due to sepsis from ARDS due to other causes, and quantitative presepsin levels were strongly correlated with SOFA score and the risk of mortality in ARDS due to sepsis.
Wen [43], 2019	Prospective cohort study	Cohort of 128 patients hospitalized due to sepsis	Presepsin correlated strongly with the SOFA score and was an independent predictor of in-hospital mortality. The combination of presepsin elevation with the SOFA score was a stronger predictor of mortality than either marker used separately.
Kondo [29], 2019	Meta-analysis	Critically ill adults from MEDLINE, EMBASE, and the Cochrane Central Register of Controlled Trials	A meta-analysis based on data from three electronic databases (MEDLINE, EMBASE, and the Cochrane Central Register of Controlled Trials) included 19 studies with 3012 critically ill adult patients aimed to evaluate the diagnostic value of procalcitonin and presepsin in the diagnosis of sepsis. It was showed that the diagnostic accuracy of both biomarkers in detecting infection was similar, also both are useful for early diagnosis of sepsis and reduction of mortality in critically ill adults.
Ugajin [51], 2019	Retrospective cohort study	172 hospitalized patients with pneumonia	The study investigated the effects of presepsin on clinical outcomes in patients with pneumonia based on the review of electronic medical records. The results showed that the patients who died had higher presepsin levels on admission. The authors concluded that in hospitalized patients with pneumonia, the plasma presepsin levels on admission could be a useful predictor of 30-day mortality.
Fujii [32], 2019	Retrospective cohort study	18 ICU patients	This retrospective study published aimed to determine the correlation between presepsin value trend and prognosis, based on ICU sepsis patients. The results showed that the 90-day mortality rate in the group of patients with decreased presepsin levels significantly improved, even when presepsin values were high on admission. The authors concluded that fifty percent decrease in presepsin levels could be served as a useful prognostic predictor of sepsis.
Hassan [35], 2019	Prospective cohort study	68 ICU patients	An observational study was conducted on 68 ICU patients with sepsis aiming to determine early predictors of sepsis-related in-hospital mortality, also to monitor the levels of presepsin and hsCRP during admission in relation to the applied treatment and the development of complications. Levels of presepsin were significantly higher at days 0 and 3 in non-survivors vs. survivors. When compared to hsCRP, presepsin was an early predictor of sepsis-related in-hospital mortality in ICU patients. The authors concluded that changes in presepsin concentrations over time may be useful for sepsis monitoring and for stratifying high-risk patients on ICU admission that benefit from intensive treatment.
Titova [50], 2018	Prospective cohort study	75 patients with sepsis due to pneumonia	This study was intended to evaluate presepsin as a marker of diagnosing severe pneumonia and sepsis. 75 patients with pneumonia, sepsis, and other inflammatory diseases were assessed and have been investigated for the level of the presepsin. All enrolled patients had obtained an antibacterial therapy in other health care centers before entering the hospital. Results showed that presepsin in patients with pneumogenic sepsis was higher compared to presepsin of patients with severe pneumonia and non-severe pneumonia. The high level of presepsin is an indication of an active infectious disease and reflects the severity of the pneumonia and development of sepsis.
Bamba [53], 2018	Prospective cohort study	11 patients hospitalized for fungal infection	This prospective study investigated the plasma levels of presepsin in fungal bloodstream infections in relation to the disease severity and in comparison to bacterial infections. The investigators assessed the associations of CRP, procalcitonin, and presepsin concentrations with the severity of fungemia. Plasma presepsin levels were found elevated in patients with fungal bloodstream infection, with a positive relation to the disease severity. They concluded that presepsin could be a useful biomarker of sepsis due to fungal infections.
Yang [30], 2018	Systematic review and meta-analysis	10 studies and 1617 patients	This systematic review was conducted to evaluate the mortality prediction value of presepsin in sepsis, a total of 10 studies and 1617 patients were included. The levels of presepsin in the first (within 24 hours) sampling were significantly lower among survivors comparing to non-survivors. The results demonstrated some mortality prediction value in presepsin in patients with sepsis.
Brodska [44], 2018	Prospective cohort study	60 patients with sepsis and SIRS	Procalcitonin, C-reactive protein, presepsin and lactate were analyzed in 60 consecutive patients with sepsis and SIRS in the study. The SOFA score was determined also daily in patients’ assessment. The results of the study showed that presepsin did not outperform the traditional biomarkers in diagnosing sepsis from SIRS and in mortality prognosis in critically ill patients. The authors concluded that presepsin may have a limited adjunct value for diagnosis and early risk stratification, performing independently of clinical illness severity.
Matera [38], 2017	Prospective cohort study	58 ICU patients	58 ICU patients with suspected sepsis were enrolled in an observational, prospective study which aimed to evaluate the role of presepsin in predicting the outcome in parallel with procalcitonin and CRP. Presepsin levels were significantly higher in non-survivors vs survivors at the same time point. Presepsin concentrations were also significantly increased in patients with positive blood cultures. The authors concluded that presepsin has an optimal prognostic and diagnostic value in patients with severe sepsis.
Kim [33], 2017	Retrospective case-series study	Case series of 157 sepsis patients	157 septic patients were enrolled in this retrospective study. Biomarkers such as procalcitonin, presepsin, galectin-3, and soluble suppression of tumorigenicity 2 were evaluated and their concentrations were analyzed in relation to the 30-day all-cause mortality. Results showed a possible prognostic utility of all four biomarkers in sepsis. This multi-biomarker approach could be a beneficial approach for an optimized management of sepsis patients.
Song [52], 2016	Prospective cohort study	61 patients with enterocutaneous fistula (ECF) and abdominal sepsis	71 patients with enterocutaneous fistula were enrolled in a study when these were diagnosed with abdominal sepsis. The aim was to evaluate the prognostic value of presepsin in ECF. Patients with higher presepsin levels had more severe intra-abdominal infection, also high risks of complications and failure of fistula closure compared with those having lower presepsin levels. The authors concluded that presepsin seems to have prognostic values for source control of abdominal sepsis and clinical courses of enterocutaneous fistula.
Klouche [36], 2016	Prospective cohort study	144 patients in 2 ICUs in France	An observational prospective study based on data from 2 ICUs in France aimed to assess the diagnostic and prognostic value of presepsin in ICU patients with severe sepsis, septic shock and severe community-acquired pneumonia. 144 patients were enrolled in the study. Presepsin and procalcitonin levels were significantly higher in septic than in non-septic patients and in patients with septic shock. The sepsis diagnostic accuracy of presepsin was not superior to that of PCT. In the patients admitted for respiratory failure, the capability of presepsin to diagnose severe community acquired pneumonia (CAP) was significantly better than PCT. Serum levels of presepsin were predictive of ICU mortality in sepsis and in CAP patients.
Enguix-Armada [34], 2016	Prospective cohort study	388 ICU patients over 12 months period	A cohort study with 388 patients admitted in the ICU over a 12-month period, assessed whether the combination of CRP, PCT, presepsin or SCD14-ST and mid-regional pro-adrenomedullin (MR-proADM) measured in the first 24 h from ICU admission could offer a better diagnostic and prognostic management of septic patients. Among the above biomarkers, PCT, MR-proADM and presepsin were found to be complementary markers in the management of septic patients when they were measured in the first 24 h after ICU admission.
Carpio [47], 2015	Prospective case-control study	123 patients with suspected infection in the ED and 123 healthy controls	The validity of presepsin in the Emergency Department setting was assessed in this study of 123 patients with suspected infection and 123 healthy individuals. Presepsin was determined on admission, after 8, 24 and 72 h. Results showed that presepsin had a similar outcome prediction on admission to the clinical scores MEDS and APACHE II. Combination of presepsin with MEDS score improved the power for outcome prediction.
Popa [46], 2015	Retrospective cohort study	95 patients with suspected infection in the ED	A retrospective study of patients with suspected infection who presented in the Emergency Department aimed to evaluate among other parameters the relationship between values of presepsin and clinical outcome. The authors concluded that presepsin had a diagnostic and early prognostic value and is an early marker of mortality in patients with sepsis.
Masson [40], 2015	Multicenter Albumin Italian Outcome Sepsis (ALBIOS) trial	997 patients with severe sepsis and septic shock	Plasma presepsin values were measured 1, 2, and 7 days in 997 patients with severe sepsis or septic shock who were enrolled in the multicenter Albumin Italian Outcome Sepsis (ALBIOS) trial. The association of single measurements of presepsin or changes over time with clinical events, organ dysfunctions, appropriateness of antibiotic therapy, and ICU or 90-day mortality were then tested. Results of the study showed that baseline presepsin was independently associated with the risk of ICU and 90-day mortality. Increasing concentrations of presepsin from day 1 to day 2 were related to higher ICU and 90-day mortality. Changes in concentrations over time seem to reflect the appropriateness of antibiotic therapy. It was concluded that presepsin seems to be an early predictor of host response and mortality in septic patients.
Sargentini [37], 2015	Prospective cohort study	21 ICU patients	Presepsin was evaluated as a potential biomarker for bacterial infection relapse in critical care patients. 21 adult patients were studied during hospitalization in an Italian Critical Care Unit. The results showed that in patients with a clinical recurrence of sepsis, while procalcitonin levels were normalized during the transient remission phase, presepsin levels remained high. The existence of maximal presepsin levels could alert clinicians not to suspend antibiotic treatments in sepsis patients.
Behnes [39], 2014	Prospective cohort study	116 ICU patients with suspected severe sepsis or septic shock	A mono-centric prospective study with 116 patients with suspected severe sepsis or septic shock aimed to evaluate the diagnostic and prognostic accuracy of presepsin in these patients during the first week of ICU treatment. The enrolled patients were followed up for six months and measurement of several biomarkers was performed. Presepsin revealed a valuable diagnostic capacity to differentiate sepsis severity compared to other used biomarkers for sepsis such as PCT, IL-6, CRP, WBC. Presepsin and IL-6 also showed prognostic value regarding 30 days and 6 months all-cause mortality throughout the first week of ICU treatment.
Endo [48], 2014	Multicenter prospective cohort study	103 patients with sepsis admitted to the ED or ICU. Also sepsis severity scores were calculated	A multicenter prospective study compared the clinical utility of presepsin with other sepsis biomarkers including procalcitonin, interleukin-6, and CRP for evaluating the severity of sepsis during follow-up. 103 patients with sepsis admitted to the ED or ICU were enrolled and classified into 3 groups: sepsis, severe sepsis and septic shock. The patients were further divided into the favorable and unfavorable prognosis groups on the basis of sepsis severity scores (i.e., SOFA and APACHE II). The patients in the favorable prognosis group had significant decreases in all biomarker levels on days 3 and 7 after admission. In the unfavorable prognosis group, only presepsin levels did not decrease significantly during follow-up. This group had significantly higher 28-day mortality than the favorable prognosis group. Presepsin levels correlated with sepsis severity during follow-up in comparison with other conventional sepsis biomarkers.
Ishikura [49], 2014	Prospective cohort study	82 patients with ≥1 SIRS criteria who were admitted in the ED. 11 biomarkers were evaluated	A study enrolled 82 patients with ≥1 SIRS criteria who were admitted in the ED, aimed to define a biomarker panel to predict sepsis-induced disseminated intravascular coagulation (DIC). 11 biomarkers were evaluated on ED admission. Among the 11 biomarkers, the optimal 2-marker panel comprised presepsin and protein C with the best accuracy of predicting sepsis. It was concluded that this biomarker panel of presepsin and protein C is predictive of the severity of sepsis-induced DIC in suspected ED patients.
Masson [41], 2014	Prospective cohort (ALBION study)	Patients with sepsis in Italian ICUs	A retrospective, case-control study of a multicenter, randomized clinical trial enrolling patients with severe sepsis or septic shock in ICUs in Italy evaluated the prognostic value of presepsin and compared it to PCT. 50 survivors and 50 non-survivors at ICU discharge, matched for age, sex and time from sepsis diagnosis were enrolled. Plasma samples were collected for 1, 2 and 7 days. Results showed that presepsin was the only variable independently associated with ICU and 28-day mortality. It also showed better prognostic accuracy than PCT in the range of SOFA score.

Presepsin and Renal Failure

Due to its relatively low molecular weight, presepsin is filtered by the glomeruli in the kidneys, where it is subsequently reabsorbed and proteolyzed in the proximal tubule. In healthy individuals, the small amounts of presepsin produced in the absence of infection are removed from circulation primarily by the glomerular filtration in the kidney. Thus it would be expected that patients suffering from renal failure (characterized by a reduction of the glomerular filtration rate) would have higher baseline presepsin concentrations. This may be particularly pronounced in dialysis-dependent individuals with end stage renal disease, as presepsin is not filtered during dialysis. In critically ill septic patients, rapidly rising presepsin levels may be indicative of acute renal failure, which is a grim but unfortunately common complication of sepsis.

A range of presepsin levels in the absence of acute pathology was established for individuals with normal renal function compared with patients with chronic renal failure. Individuals with grade 3, 4 and 5 renal failure had baseline presepsin values significantly above the other group, with values of 208.1 ± 70.2 pg/mL, 320.2 ± 170.1 pg/mL, 712.8 ± 336.3 pg/mL, respectively [54]. Three studies on patients presenting with acute kidney injury and sepsis indicated that in the validity of presepsin measurement in this population was reduced [55, 56] and procalcitonin was superior as a diagnostic and prognostic marker of sepsis in this group [57]. The authors however noted that presepsin could also be used, with the caveat that a different reference range for values be utilized and its use be limited to less severe presentations of acute renal failure (Table 3).

**Table 3:** Studies on presepsin measurement interpretation in renal failure AKI: Acute kidney injury, pg/ml: picograms per millilitre, PCT: procalcitonin, RIFLE: Risk, Injury, Failure, Loss of kidney function, and End-stage kidney disease.

1^st^ Author and year of publication	Study design	Type of patients/database	Major findings
Miyoshi [54], 2019	Cross-sectional	Study on the effect kidney function on presepsin levels in healthy volunteers (47) and individuals with chronic renal failure (85)	A range of presepsin levels in the absence of acute pathology was established for individuals with normal renal function compared with patients with chronic renal failure. Individuals with grade 3, 4 and 5 renal failure had baseline presepsin values significantly above the other group, with values of 208.1 ± 70.2 pg/mL, 320.2 ± 170.1 pg/mL, 712.8 ± 336.3 pg/mL, respectively.
Nakamura [55], 2019	Prospective cohort study	806 patients with/without acute kidney injury (AKI)	The study assessed the validity of presepsin and procalcitonin plasma levels for diagnosing sepsis in patients with and without acute kidney injury. Each group of patients was subdivided according to sepsis status for each stage of the kidney injury. The results showed that for patients with severe acute kidney injury, the accuracy of the diagnosis of sepsis with procalcitonin was significantly higher compared to that with presepsin.
Takahashi [56], 2015	Retrospective case-control study	91 patients with/without acute kidney injury (AKI)	In a retrospective study with 91 patients, the diagnostic accuracy of PCT and presepsin was assessed when patients were divided into two groups with/without acute kidney injury (AKI). PCT and presepsin levels were increased significantly in the non-AKI and AKI patients with infection. It was concluded that PCT and presepsin are useful markers of bacterial infections in AKI but different thresholds should be applied.
Nakamura [57], 2014	Single center retrospective case-control study	247 ICU patients with/without acute kidney injury (AKI)	A single center retrospective study referred to 247 patients admitted to the ICU aimed to determine levels of blood presepsin in patients with or without sepsis and among non-AKI patients or patients with different degrees of acute kidney injury (AKI) disease. Patients were classified into non-AKI and AKI according to the RIFLE criteria. It was finally showed that presepsin level can be a reliable indicator of sepsis, also in sepsis patients with less severe forms of AKI.

Discussion

Early diagnosis and timely treatment are the corner stones of survival improvement in sepsis syndrome. There is still a need for identifying the ‘ideal’ biomarker as none of the common applied in clinical practice such as the white blood cell count (WBC), procalcitonin (PCT), C-reactive protein (CRP) and interleukin-6 (IL-6), has a 100% sensitivity and specificity. Apart from these commonly used biomarkers, many attempts of using molecules which are participating in the sepsis cascades failed to identify the most appropriate one in clinical practice.

CD14, a glycoprotein expressed on monocytes and macrophages, is part of the innate immune system serving as a receptor for lipopolysaccharides (LPS) of bacteria and activating the pro-inflammatory signaling cascade. CD14 molecule is a pattern recognition receptor existing in two forms: a membrane-bound type (mCD14) and a soluble form (sCD14). Both forms play a role in recognition of bacteria LPS and in cell activation. During the progression of the sepsis cascades where the soluble CD14 fragments are cleaved, the soluble CD14 subtype (sCD14-ST) also called as presepsin elevates significantly and is readily measured using a chemiluminescent enzyme immunoassay. It is a small 13 kDa protein that arises due to cleavage of the N-terminal fragment of CD14 by elastase [2]. Presepsin is filtered through the glomeruli, then reabsorbed, and catabolized within the proximal tubular cells. Presepsin should be interpreted attentively in patients with kidney disease, as elevated presepsin levels were found in patients with decreased renal function, also inverse correlation has been described between presepsin and GFR as well [58,59].

This molecule is now recognized as a new sepsis biomarker and can also be used in the differentiation between bacterial infections and non-infectious SIRS [25]. Presepsin is normally present in very low concentrations in the serum of healthy individuals. In response to bacterial infections, its concentration increases within 2 hours, according to the severity of the disease and the cut-off levels for sepsis have been reported between 400-600 pg/ml. The early increase in levels of presepsin during the sepsis cascade and other bacterial infections have made it an attractive indicator for laboratory testing.

The findings of this review indicate that presepsin is a molecule that has been used in clinical practice the latest years in an attempt to evaluate patients suffering from sepsis. Among the extracted bibliography, there are some studies which do not indicate a superiority of presepsin in regards to the already used biomarkers [12,29,37]. However, there are studies which propose presepsin as a highly sensitive and specific marker of sepsis, as its concentration significantly correlates with the severity of sepsis syndrome and in-hospital mortality [1,2,39,41]. Furthermore, some studies provide preliminary evidence that in certain settings presepsin may be a superior diagnostic and prognostic biomarker of sepsis compared to the more common biomarkers CRP and PCT [8,22,60,61]. Other studies also emphasize the role of presepsin in the assessment of sepsis in combination with commonly used biomarkers and clinical rating scales, as part of a multi-biomarker approach of sepsis in view of the absence of the ‘ideal’ biomarker [6,13,28,33]. Presepsin is a promising biomarker for the evaluation of sepsis both in the emergency department to aid in the initial assessment of the patient and in the intensive care setting where patients are most likely to be in a state of multiple organ failure [34,39,40,62].

Although a strong body of literature favors the validity of presepsin as a biomarker for the diagnosis, prognosis and risk stratification of sepsis, presepsin measurement is not yet ubiquitously available as a routine laboratory test. The studies in favor of the use of presepsin are heterogenous, examining different patient groups at risk of sepsis in a variety of different settings, with the small sample size being a common limitation. Presepsin does not appear to be clearly superior to the biomarkers commonly used in the assessment of sepsis, but may be valuable when used in conjunction with other established tests to better identify patients at risk of clinical deterioration. Further studies are indicated to establish whether it is useful for predicting the most severe complications of sepsis in the intensive care setting, and the evidence regarding its use in the postoperative setting also warrants a careful appraisal.

## Conclusions

Presepsin has some value in the evaluation of the severity and prognosis of sepsis and as the results of the test are available within 2 hours, widespread clinical use is feasible, though it is unclear at this time whether it would significantly affect clinical practice. As sepsis syndrome remains an entity with high mortality rates and increased socioeconomic implications and the ‘ideal’ biomarker has not yet been identified, further research is warranted to evaluate the role of presepsin alone or in combination with other biomarkers in the assessment of sepsis.
